# Demonstration of multiple-wavelength-band photonic integrated circuits using a silicon and silicon nitride 2.5D integration method

**DOI:** 10.1515/nanoph-2025-0234

**Published:** 2025-12-15

**Authors:** Meicheng Fu, Huaqing Qiu, Hongyu Zhang, Xin Chen, Junli Qi, Yi Zhang, Yao Xu, Siyu Liu, Nan Gu, Hongtao Yu, Wenjun Yi, Xiujian Li, Xiaowei Guan

**Affiliations:** College of Science, 58294 National University of Defense Technology, 410073, Changsha, China; DTU Electro, Department of Electrical and Photonics Engineering, Technical University of Denmark, DK-2800, Kgs. Lyngby, Denmark; Jiaxing Key Laboratory of Photonic Sensing & Intelligent Imaging, Intelligent Optics & Photonics Research Center, Jiaxing Research Institute, Zhejiang University, Jiaxing, 314000, China; Centre for Optical and Electromagnetic Research, College of Optical Science and Engineering, Zhejiang University, Hangzhou, 310058, Zhejiang, China

**Keywords:** photonic integrated circuits, 2.5D integration, all-optical modulator, micro-ring resonator

## Abstract

Conventional photonic integrated circuits (PICs) are fundamentally limited by single-wavelength-band operation. To transcend this barrier, we introduce a multiple-wavelength-band platform using a 2.5D integration scheme that monolithically combines silicon and silicon nitride waveguides side-by-side on a single chip. This architecture natively supports simultaneous 850 nm and 1,550 nm transmission while eliminating key limitations of 3D integration such as chemical-mechanical polishing and fixed coupling gaps. As a critical demonstration, we realize an all-optical modulator where 850 nm pump light controls a 1,550 nm signal in a silicon microring resonator, achieving a record-high modulation efficiency of −0.023 nm/mW and 93 % depth – surpassing existing schemes. This work establishes a scalable pathway beyond single-band PICs, opening new frontiers in programmable photonics and on-chip signal processing, etc.

## 1 Introduction

Photonic integrated circuits (PICs) have enabled a plethora of fascinating applications ranging from optical communications to quantum technologies, matter analysis, microwave photonics and so on [1], [2], [3], [4], [5], [6]. Conventionally, for a specific sort of applications, the PIC chips only operate in a specific wavelength band. For example, PICs for the long-haul optical communications operate at the wavelengths around 1,550 nm [7], [8], [9], [10], [11], while the short ones prefer the wavelengths around 850 nm [12]. Quantum information processing PIC chips generally use the visible lights [13], [14], [15] while the mid-infrared wavelengths are the preferred choice when considering the on-chip gas sensors [16], [17], [18], [19].

In spite of currently being the workhorse in PICs, the conventional single-wavelength-band paradigm imposes fundamental limitations on PICs. It suffers from material inefficiency, as a single waveguide is often suboptimal for a broad spectrum, and crucially lacks the native capability for efficient inter-band optical interactions, which forces critical functions like all-optical modulation to rely on inefficient nonlinear effects within the same waveguide or bulky off-chip systems. To transcend these barriers, we introduce the concept of multiple-wavelength-band (MWB) PICs. This platform, by monolithically integrating distinct, optimized waveguide systems on a single chip, enables low-loss transmission for each band and natively facilitates direct and efficient cross-band control. Thereby, MWB PICs exploit the distinct specialties of all wavelength bands within one chip, which significantly improves functional density and outperforms conventional table-top or fiber-based optical circuits in terms of device footprint, manufacturing complexity, and energy consumption – ultimately unlocking new frontiers in multi-spectral sensing, programmable photonics, etc. Furthermore, the recently blooming on-chip supercontinuum generation (SCG) technology, that can generate light spectra covering multiple wavelength bands in a waveguide [20], [21], [22], provides the key light sources and thus new opportunities for developing the MWB PICs. Indeed, some prior works have shown the transmission of different bands on the same chip, such as using a common grating coupler for different bands [23], [24] or achieving on-chip optical amplification using two bands, but these implementations rely on transmission within the same waveguide [25].

For MWB PICs, heterogeneous integration of distinct waveguide systems on a single chip, allowing each wavelength band to propagate through its optimally designed waveguide, is necessary. However, this co-integration faces significant challenges in material compatibility and fabrication processes. Conventional 3D integration schemes [26], [27] suffer from inherent limitations including the requirement of dummy structures for planarization uniformity and fixed vertical coupling gaps across the wafer. Here, we introduce a 2.5D integration methodology that fundamentally transforms the integration paradigm from vertical stacking to planar side-by-side architecture. This approach eliminates the need for chemical mechanical polishing while enabling device-specific coupling gaps, establishing a new framework for multi-band photonic integration.

To validate this platform, we demonstrate an all-optical modulator capable of simultaneous 850 nm and 1,550 nm operation. Using the 2.5D integration approach, silicon nitride (SiN) waveguides are fabricated alongside silicon microring resonators (MRRs). Pump light at 850 nm propagating through the SiN waveguide is absorbed by the adjacent silicon, generating free carriers that dynamically modulate the resonance of the 1,550 nm signal in the MRR. Static and dynamic measurements demonstrate an all-optical modulation efficiency of −0.023 nm/mW and a modulation depth of 93 %. These results surpass the performance of conventional single-wavelength-band schemes and out-of-plane control methods, confirming the effectiveness of the proposed integration strategy for advanced multi-band photonic systems.

## 2 Results and discussion

### 2.1 Principle and fabrication

**Figure 1:**
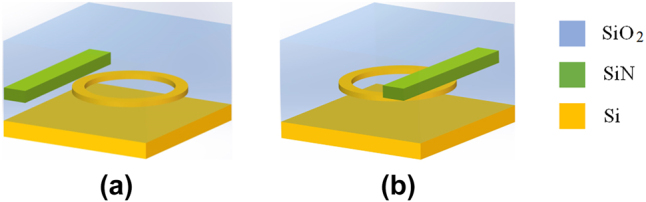
Schematic views of the proposed 2.5D integration in this work (a) and the 3D integration with the deposition of the SiN film on the Si waveguides (b).

The proposed on-chip all-optical modulator based on the 2.5D Si–SiN integration method can be schematically illustrated in Figure 1a. In the modulator, the SiN waveguide is located beside the Si waveguide MRR to allow the light transmitting in the SiN waveguide to modulate the MRR. As comparisons, we also show schematic of the conventional 3D integration method in Figure 1b, where the Si devices are first fabricated and then the SiN waveguides are fabricated on top of the Si waveguide layer. Si–SiN integrated devices including the couplers have been demonstrated using the 3D integration method [28], [29], [30].

**Figure 2:**
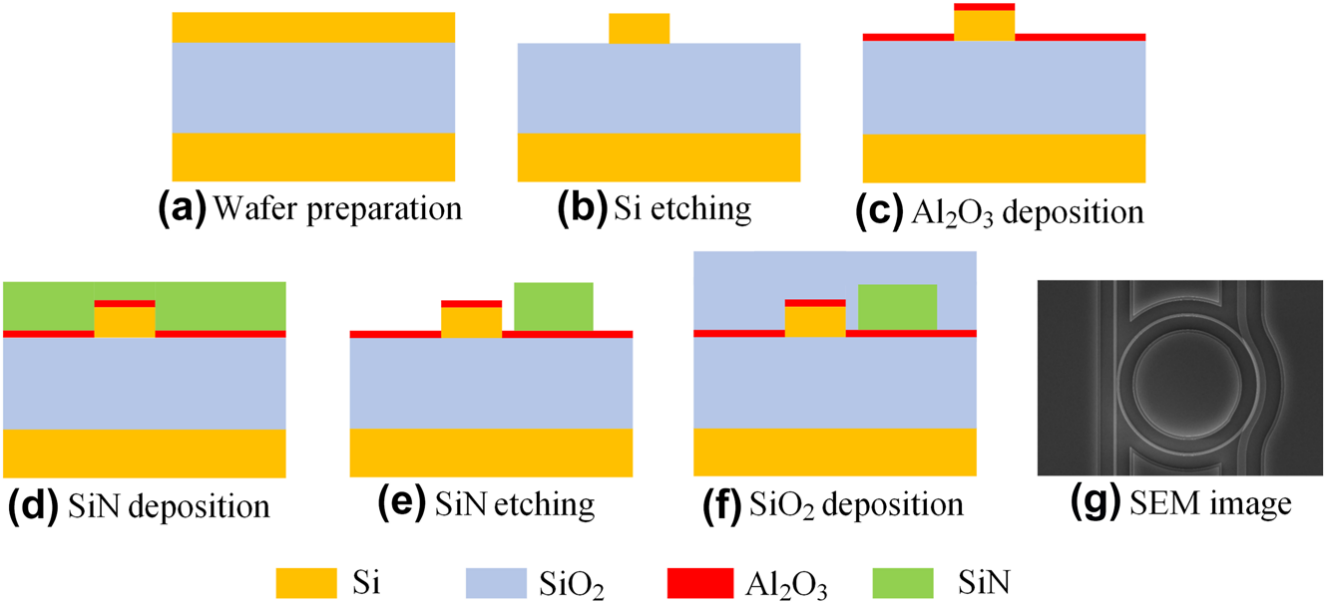
Fabrication processes of the Si–SiN 2.5D integration chip (a–f) and the SEM image of the all-optical modulator on the chip (g).

The fabrication flow of the proposed 2.5D integration chip is shown in Figure 2. Firstly, a standard silicon-on-insulator (SOI) fabrication process, including the E-beam lithography (EBL) and the reactive-ion etching (RIE), is applied to fabricate the Si MRR (Figure 2a and b). Then, a thin aluminium oxide (Al_2_O_3_) film is deposited using the atomic layer deposition (ALD) as shown in Figure 2c, which will act as the protective layer during the SiN etching. Next, the SiN waveguide is deposited using the plasma-enhanced chemical vapor depostion (Figure 2d) and dry etched with an EBL resist as the mask (Figure 2e). It is noteworthy that this step involves overlay alignment, with a precision of around 10 nm. Finally, a SiO_2_ film cladding layer is deposited as shown in Figure 2f. Figure 2g shows the scanning electron microscopy (SEM) image of the fabricated all-optical modulator on the chip. In our case, the thicknesses of the Si, SiN and Al_2_O_3_ are 250 nm, 400 nm and 20 nm, respectively. It should be noted that this is a general fabrication flow for the proposed 2.5D integration and not limited to the Si–SiN materials or the waveguide dimensions shown in this work.

As evident from the above process flow, the 2.5D approach presented in this work eliminates the chemical mechanical polishing (CMP) step, which is indispensable in 3D integration. Thereby, it avoids the need for numerous dummy structures in the lower silicon waveguide layer – essential in 3D integration to ensure CMP uniformity through pattern density modulation – greatly simplifying waveguide and device layout. Furthermore, while 3D integration fixes the vertical coupling gap uniformly across the wafer and risks polarization rotation with lateral misalignment, the 2.5D scheme employs lateral coupling, allowing the gap to be freely adjusted per device and significantly enhancing design flexibility.

### 2.2 Static measurements

**Figure 3:**
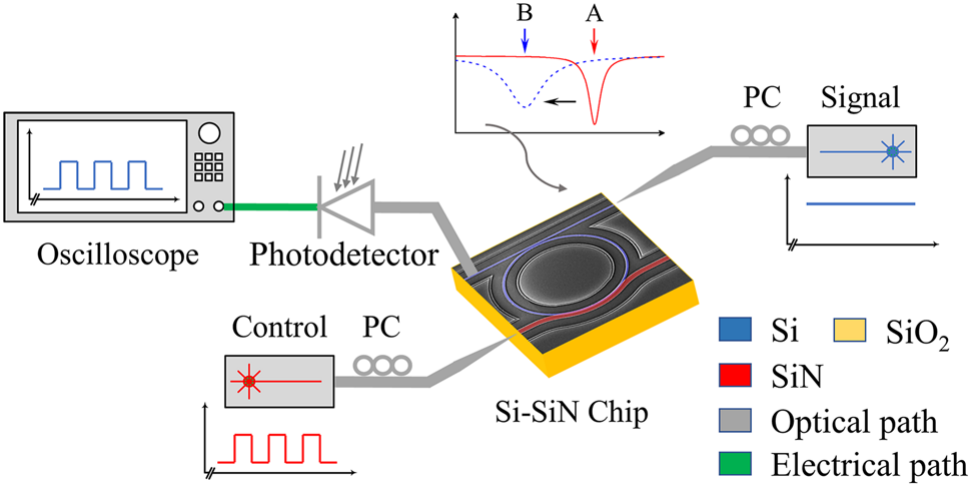
Experimental setup for measuring the MWB all-optical modulator using the 2.5D Si–SiN integration. The SEM image with fake colors highlights the key parts with red for the SiN waveguide and blue for the Si MRR.

The experimental setup of the MWB all-optical modulator is shown in Figure 3. A tunable laser source (TLS) is used to inject the signal light into the Si waveguide through a lensed fiber, while the output signal is coupled to a standard single mode fiber (SMF) by a grating coupler. The combination of a photodetector and an oscilloscope is used to record the signal. The control light is injected into the SiN waveguide through a lensed fiber as well. The working principle of the proposed all-optical modulator can be explained as below. The control light transmitting in the SiN waveguide and possessing wavelength of 850 nm, which is within the absorption band of Si, can be absorbed by the Si waveguide to generate the free carriers when it passes through the Si–SiN coupled waveguide region. The free carriers can shift the resonance wavelength towards the short wavelength and degrade the intrinsic *Q* values of the Si MRR through the free-carrier dispersion (FCD) and the free-carrier absorption (FCA), respectively. The change from the red solid line to the blue dashed line examplifies one case of the principle. In the case, the original operating mode of the MRR is around the critical coupling with a large extinction ratio when the control light is off (A). While after the control light is turned on, the MRR operates at the under-coupling mode (B). Consequently, the 1550-nm-wavelength signal light transmitting in the silicon waveguide is modulated by the control light.

**Figure 4:**
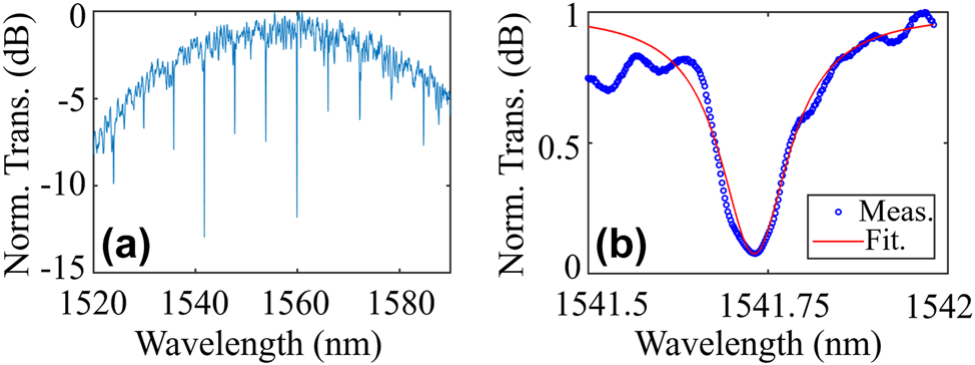
(a) Normalized transmission spectrum of the under-test MWB all-optical modulator and (b) zoom-in view around 1,541 nm with the Lorentz fitting.

The device under-test (DUT) is first characterized by sweeping the tunable laser source (TLS) wavelengths and recording the transmission spectra. Figure 4a shows the normalized (to the maximal power of the spectrum) transmission spectrum and Figure 4b is a zoom-in view of the spectrum around 1,541 nm. Lorentz fitting is applied, giving an extracted intrinsic *Q* value (*Q*_int_) of 3.6 × 10^4^.

**Figure 5:**
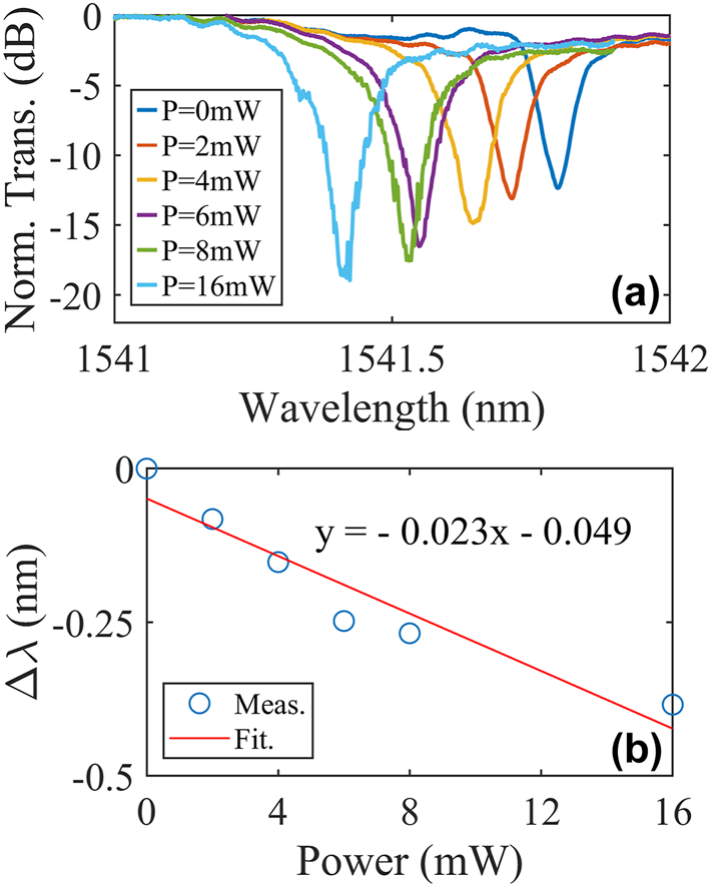
Measured static responses of the MWB all-optical modulator at different powers of the control light with the wavelength of 850 nm. (a) Normalized transmission spectra. (b) Measured (blue circle) and fitted (red line) resonance shifts versus the powers.

The static measurements with a control light wavelength of 850 nm are then implemented and the results are shown in Figure 5. Figure 5a shows the resonance wavelength blue shifting with the control light power increasing (the power in this work refers to that in the lensed fiber). Apparently, the resonance shifts to the shorter wavelengths and the extinction ratios (ERs) increase as well, indicating the over-coupling of the under-test Si MRR at the original state. Figure 5b is the measured resonance shift with respect to the control light powers and the linear fitting, from which a tuning efficiency of −0.023 nm/mW is extracted.

**Figure 6:**
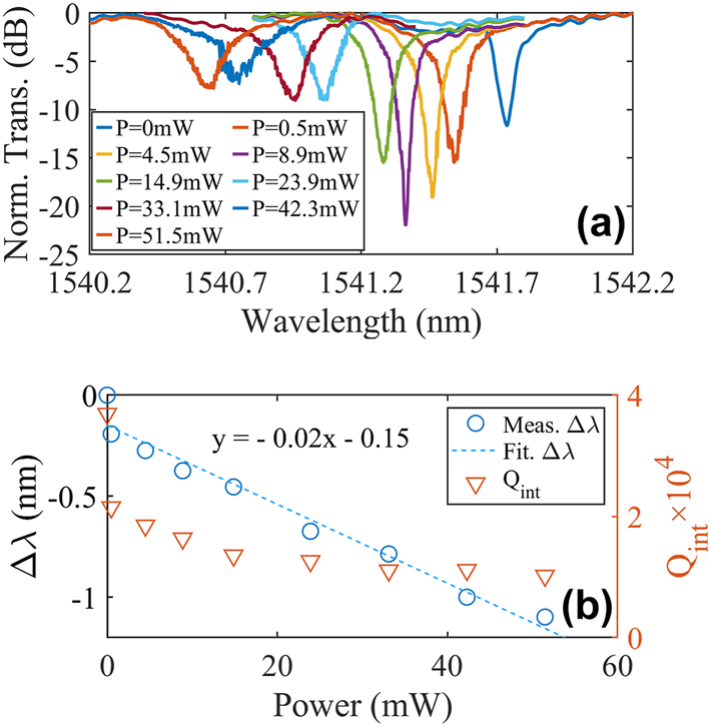
Measured static responses of the MWB all-optical modulator at different powers of the control light with the wavelengths around 980 nm. (a) Normalized transmission spectra. (b) Measured (blue circle) and fitted (dashed blue line) resonance shifts and the intrinsic Qs (orange triangular) versus the powers.

In order to further explore the performances of the all-optical modulator, a laser with the wavelengths around 980 nm is used as the control and the measurement results are shown in Figure 6. Since the power of this laser can be much larger, one can clearly find the coupling condition of the MRR is changed from over-coupling to under-coupling with the ERs first increase and then decrease. The *Q*_int_ and resonance shift as a function of the control light powers extracted from Figure 6a are shown in Figure 6b. A tuning efficiency of −0.02 nm/mW is also achieved, suggesting that the modulator is insensitive to the wavelength of the control light. It is worthy to highlight that the tuning efficiency achieved here is around five times larger than that in Ref. [31]. Thanks to the 2.5D integration, the control light can be more efficiently coupled and absorbed by the silicon waveguide on the chip. That is why the tuning efficiency is greatly magnified. The *Q*_int_ is also extracted as shown in Figure 6b and the results show clear degeneration caused by FCA as the control light powers increase.

### 2.3 Dynamic measurements

**Figure 7:**
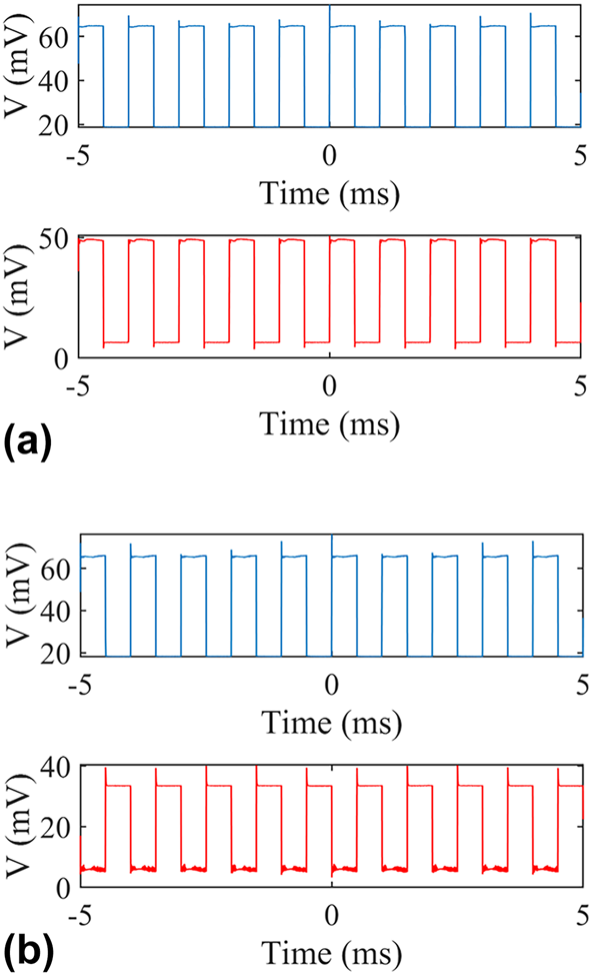
Measured dynamic responses of the MWB all-optical modulator with an 850-nm control light. Blue (input control light) and red (output signal light) lines illustrate the data extracted from the oscilloscope. Wavelength of the signal light is set for the original signal at the “off” state (a) and the “on” state (b).

The dynamic responses of the MWB all-optical modulators are characterized and the results extracted from the oscilloscope are shown in Figure 7, where the blue line represents the input control light while the red line represents the output signal light. The 850-nm control light laser with a repetition of 1 kHz, a duty cycle of 50 % and an average power of 7 mW is used. The wavelength of the signal light is set different, where the original signal is “off” in Figure 7a and “on” in Figure 7b. It is clearly shown that the control light can dynamically switch the all-optical modulator no matter the original state. Modulation depth (MD) is a widely used as a figure-of-merit to evaluate the performance of a modulator, and MD is defined as follows.

(1) $$\text{MD}=\left(I_{\max}-I_{\min}\right)/I_{\max}$$

Here, *I*_max_ is the maximum transmission of the signal while *I*_min_ is the minimum. The MD is calculated to be 93 % and 92 % in Figure 7a and b, respectively.

### 2.4 Discussion and conclusion

As a comparison, we have summarized the performances of the MRR-based all-optical modulators using different schemes, as listed in Table 1. In [32], a pulsed pump light was used to control the signal light transmitting in the same Si MRR. In this in-plane scheme, both lights had wavelengths around 1,550 nm and the carriers generated from the two-photon absorption (TPA) played as the controlling media. Since the maximal wavelength shift was −0.36 nm at a peak power of 2.35 W in the input fiber (Gaussian-type pulse train with repetition rate of 78 MHz, pulse duration of 10 ps and pulse energy of 25 pJ), the tuning efficiency was about −1.5 × 10^−4^ nm/mW. With the similar in-plane scheme but leveraging the Kerr effect in a Si slot MRR, Martınez et al. [16] demonstrated an all-optical modulator exhibiting an efficiency of 3.1 × 10^−4^ nm/mW, given that the maximal wavelength shift was about 0.5 nm at the peak power of the pulsed pump in the input fiber of about 1,600 mW (Gaussian-type pulse train with repetition rate of 10 GHz, pulse duration of 10 ps and pulse energy of 10 pJ). Despite using the in-plane scheme, these two demonstrations show modulation efficiency about two orders smaller than that proposed in this work, since they only operated in one wavelength band and the inefficient third order nonlinear effects had to be involved. On the other hand, even if multiple wavelength bands were involved but with the out-plane scheme, the modulation efficiency could be still low. This happened in the case where an ultra-violet pulsed pump light illuminated onto the top surface of a silicon MRR [33]. Due to the very strong reflection and thus the highly inefficient one-photon absorption (OPA), the modulation efficiency was only −2.9 × 10^−7^ nm/mW. Here, this calculation used a maximal wavelength shift of −1.1 nm under a peak power of 376 W (Gaussian-type pulse train with repetition rate of 80 MHz, pulse duration of 100 fs and pulse energy of 40 pJ). Rather than taking advantage of the generated carriers as the controlling media, Wei et al. introduced the photothermal effect to realize the all-optical modulation [31]. The 980-nm-wavelength light illuminated onto a two-dimensional material (PtSe_2_) which was connected to a Si MRR and could conduct the generated thermal energy to the Si MRR. Though this could dramatically improve the modulation efficiency to −4 × 10^−3^ nm/mW, as an out-plane scheme, it was still less efficient than the proposed work. All in all, from the Table 1, one can clearly see our proposed MWB all-optical modulator using the 2.5D integration is superior to the already reported methods. Meanwhile, the new method can still sustain very high modulation depth.

**Table 1:** Performance summary of the MRR-based all-optical modulators using different schemes.

Materials	*λ*_signal_ & *λ*_control_ (nm)	MD	Efficiency (nm/mW)	Coupling	Principles
Si MRR [32]	1,535.2, 1,555.5	94 %	−1.5 × 10^−4^	In-plane	TPA-carriers
Si MRR [33]	1,555.5, 400	97 %	−2.9 × 10^−7^	Out-plane	OPA-carriers
Si Slot MRR [16]	1,544.5, 1,557.5	56 %	3.1 × 10^−4^	In-plane	Kerr effect
PtSe_2_-clad Si MRR [31]	1,550, 980	60 %	−4 × 10^−3^	Out-plane	Photothermal effect
2.5D Si–SiN MRR (this work)	1,541, 850/980	93 %/92 %	(−2.3/−2) × 10^−2^	In-plane	OPA-carriers

**Figure 8:**
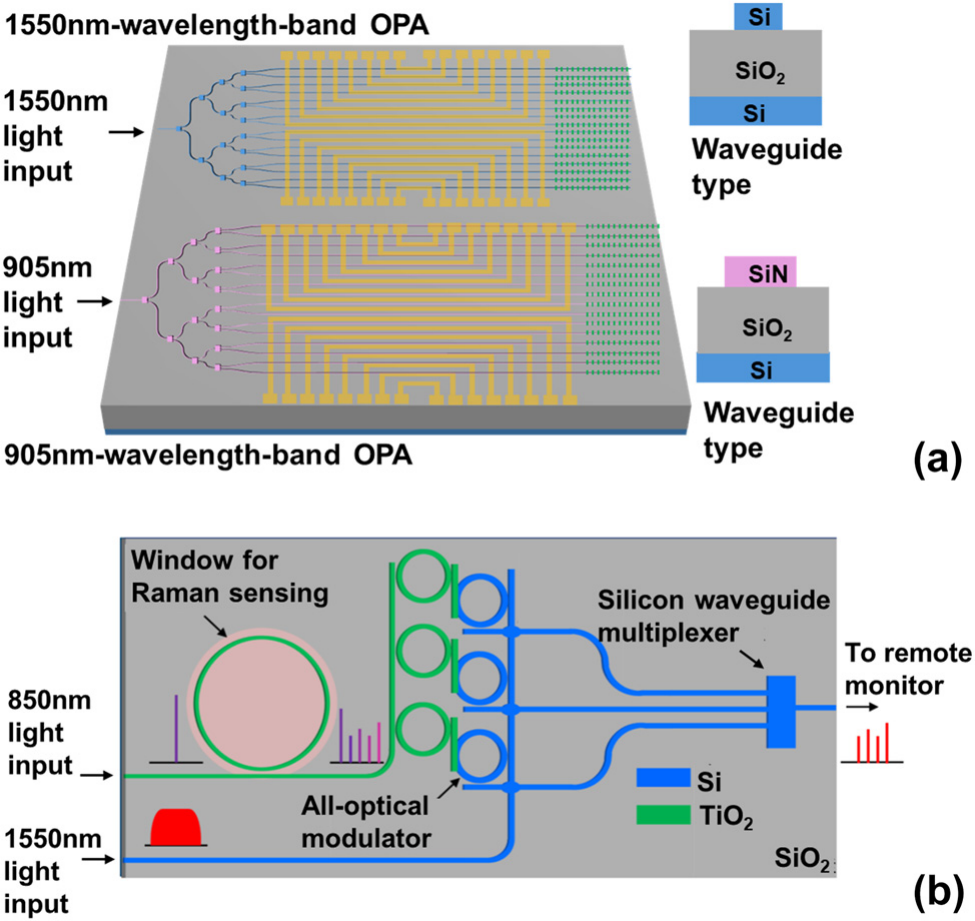
Conceptual schematics for advanced applications using the demonstrated MWB PIC with the novel 2.5D integration method. (a) Dual-band on-chip OPA. (b) Integrated communication and sensing chip.

Furthermore, to demonstrate the practical potential of our 2.5D integrated MWB PIC platform, we envision two promising applications, as illustrated in Figure 8. Figure 8(a) shows a dual-band optical phased array (OPA) for LiDAR, which monolithically integrates 1,550 nm and 905 nm operational bands using dedicated Si and SiN waveguides, respectively. This heterogeneous integration overcomes the inherent limitations of single-material systems – enabling simultaneous exploitation of the eye-safe, high-power advantages at 1,550 nm and the mature, low-cost ecosystem at 905 nm. Figure 8(b) depicts an integrated communication and sensing chip, where a titanium dioxide (TiO_2_) waveguide performs Raman sensing at 850 nm, and the resulting signal is all-optically modulated onto a 1,550 nm carrier in an adjacent silicon waveguide for remote transmission. This architecture facilitates fully optical sensing operation in electromagnetic-sensitive environments without signal electro-optic conversion. Together, these examples highlight the flexibility of our platform in supporting advanced multi-band and multi-functional photonic systems that transcend the capabilities of conventional single-wavelength-band designs. In conclusion, to break free from the conventional paradigm of single-wavelength-band photonic integrated circuits, we introduce a multiple-wavelength-band platform based on a 2.5D integration strategy. This planar integration method enables the simultaneous incorporation of optically disparate waveguide systems – each tailored to a specific wavelength band – overcoming the material and structural limitations inherent in 3D integration. By avoiding chemical mechanical polishing and permitting device-specific coupling design, the platform significantly enhances layout flexibility and process simplicity. As a functional demonstration, we realized an all-optical modulator achieving a record efficiency of −0.023 nm/mW and 93 % modulation depth, confirming the platform’s ability to support efficient cross-band optical control. This study thereby expands the scope of integrated photonics beyond single-band operation and provides a scalable foundation for future programmable photonics, multi-spectral sensing, and high-capacity on-chip signal processing.

## References

[1] Hao Y. (2021). Recent progress of integrated circuits and optoelectronic chips. *Sci. China Inf. Sci.*.

[2] Heck M. J., Bauters J. F., Davenport M. L., Spencer D. T., Bowers J. E. (2014). Ultra-low loss waveguide platform and its integration with silicon photonics. *Laser Photon. Rev.*.

[3] Wang J. (2018). Multidimensional quantum entanglement with large-scale integrated optics. *Science*.

[4] Tran M. A. (2022). Extending the spectrum of fully integrated photonics to submicrometre wavelengths. *Nature*.

[5] Sibson P., Kennard J. E., Stanisic S., Erven C., O’Brien J. L., Thompson M. G. (2017). Integrated silicon photonics for high-speed quantum key distribution. *Optica*.

[6] Chen H., Cao H., Yu Z., Zhao W., Dai D. (2023). Waveguide-integrated optical modulators with two-dimensional materials. *J. Semicond.*.

[7] Jørgensen A. (2022). V. Torres-company, lk oxenløwe. *Nat. Photon.*.

[8] Guan P. (2018). Scalable WDM phase regeneration in a single phase-sensitive amplifier through optical time lenses. *Nat. Commun.*.

[9] Geng Y. (2022). Coherent optical communications using coherence-cloned kerr soliton microcombs. *Nat. Commun.*.

[10] Hu H., Oxenløwe L. K. (2021). Chip-based optical frequency combs for high-capacity optical communications. *Nanophotonics*.

[11] Dong Y., Zhang Y., Shen J., Xu Z., Zou X., Su Y. (2022). Silicon-integrated high-speed mode and polarization switch-and-selector. *J. Semicond.*.

[12] Kumari S., Haglund E. P., Gustavsson J. S., Larsson A., Roelkens G., Baets R. G. (2018). Vertical-cavity silicon-integrated laser with in-plane waveguide emission at 850 nm. *Laser Photon. Rev.*.

[13] He J. (2020). Nonlinear nanophotonic devices in the ultraviolet to visible wavelength range. *Nanophotonics*.

[14] Liang G. (2021). Robust, efficient, micrometre-scale phase modulators at visible wavelengths. *Nat. Photonics*.

[15] Lu T.-J. (2018). Aluminum nitride integrated photonics platform for the ultraviolet to visible spectrum. *Opt. Express*.

[16] Martínez A. (2010). Ultrafast all-optical switching in a silicon-nanocrystal-based silicon slot waveguide at telecom wavelengths. *Nano Lett.*.

[17] Kou R. (2018). Mid-ir broadband supercontinuum generation from a suspended silicon waveguide. *Opt. Lett.*.

[18] Singh N. (2015). Midinfrared supercontinuum generation from 2 to 6 *μ*m in a silicon nanowire. *Optica*.

[19] Schliesser A., Picqué N., Hänsch T. W. (2012). Mid-infrared frequency combs. *Nat. Photon.*.

[20] Kuyken B., Billet M., Leo F., Yvind K., Pu M. (2020). Octave-spanning coherent supercontinuum generation in an algaas-on-insulator waveguide. *Opt. Lett.*.

[21] Yu M., Desiatov B., Okawachi Y., Gaeta A. L., Lončar M. (2019). Coherent two-octave-spanning supercontinuum generation in lithium-niobate waveguides. *Opt. Lett.*.

[22] Mouawad O. (2014). Multioctave midinfrared supercontinuum generation in suspended-core chalcogenide fibers. *Opt. Lett.*.

[23] González-Andrade D. (2021). Dual-band fiber-chip grating coupler in a 300 mm silicon-on-insulator platform and 193 nm deep-UV lithography. *Opt. Lett.*.

[24] Hao T., Sánchez-Postigo A., Cheben P., Ortega-Moñux A., Ye W. N. (2020). Dual-band polarization-independent subwavelength grating coupler for wavelength demultiplexing. *IEEE Photon. Technol. Lett.*.

[25] Liu Y. (2022). A photonic integrated circuit–based erbium-doped amplifier. *Science*.

[26] Bauters J. F. (2013). Silicon on ultra-low-loss waveguide photonic integration platform. *Opt. Express*.

[27] Sacher W. D. (2018). Monolithically integrated multilayer silicon nitride-on-silicon waveguide platforms for 3-D photonic circuits and devices. *Proc. IEEE*.

[28] Sodagar M., Pourabolghasem R., Eftekhar A. A., Adibi A. (2014). High-efficiency and wideband interlayer grating couplers in multilayer Si/SiO_2_/SiN platform for 3D integration of optical functionalities. *Opt. Express*.

[29] Huang Y., Song J., Luo X., Liow T.-Y., Lo G.-Q. (2014). CMOS compatible monolithic multi-layer Si_3_N_4_-on-SOI platform for low-loss high performance silicon photonics dense integration. *Opt. Express*.

[30] Sacher W. D. (2014). Wide bandwidth and high coupling efficiency Si_3_N_4_-on-SOI dual-level grating coupler. *Opt. Express*.

[31] Wei K. (2020). All-optical PtSe_2_ silicon photonic modulator with ultra-high stability. *Photon. Res.*.

[32] Almeida V. R., Barrios C. A., Panepucci R. R., Lipson M. (2004). All-optical control of light on a silicon chip. *Nature*.

[33] Almeida V. R. (2004). All-optical switching on a silicon chip. *Opt. Lett.*.

